# Adapting ENABLE for patients with advanced cancer and their family caregivers in Singapore: a qualitative formative evaluation

**DOI:** 10.1186/s12904-021-00799-y

**Published:** 2021-06-22

**Authors:** Grace Meijuan Yang, J. Nicholas Dionne-Odom, Yi Han Foo, Ariel Hui Mei Chung, Nur Haidah Ahmad Kamal, Laurence Tan, Chou Chuen Yu, Le Mai Khanh, Audrey Rui Xuan Koh, Irene Teo, Sungwon Yoon, Marie Bakitas

**Affiliations:** 1 National Cancer Centre Singapore, 11 Hospital Crescent, Singapore, 169610 Singapore; 2 The University of Alabama At Birmingham, Birmingham, USA; 3 National University of Singapore, Singapore, Singapore; 4 Khoo Teck Puat Hospital, Singapore, Singapore; 5 Geriatric Education and Research Institute, Singapore, Singapore; 6 Duke-NUS Medical School, Lien Centre for Palliative Care, Singapore, Singapore; 7 Duke-NUS Medical School, Singapore, Singapore

**Keywords:** Palliative Care, Patient participation, Caregivers, Culturally competent care, Qualitative research

## Abstract

**Background:**

ENABLE (Educate, Nurture, Advise, Before Life Ends) is a nurse coach-led, early palliative care model for patients with advanced cancer and their family caregivers. Content covered includes problem-solving, advance care planning, symptom management and self-care. The aim was to evaluate the cultural acceptability of ENABLE among patients with advanced cancer and their caregivers in Singapore and identify modifications for an adapted ENABLE-SG model.

**Methods:**

Qualitative formative evaluation with a thematic analysis approach in two hospitals in Singapore, involving patients (*n* = 10), family caregivers (*n* = 11) and healthcare professionals (*n* = 10) who care for patients with advanced cancer. Semi-structured interviews were conducted to explore (i) the main needs and challenges facing individuals with advanced cancer and their family caregivers; (ii) patient involvement in healthcare decision making; and (iii) content and delivery of ENABLE.

**Results:**

While physical needs were largely well met, participants expressed that psychosocial care was delivered too late in the illness trajectory. Healthcare decision making approaches varied from a patient-centred shared decision-making model to a family-centred model where patients may not know their cancer diagnosis and prognosis. The content was considered to be relevant, comprehensive and practical; financial assistance, adjustment to body image, and evaluation of complementary therapy were also recommended. Face-to-face rather than telephone sessions were preferred to facilitate rapport building.

**Conclusions:**

ENABLE was broadly acceptable with some modifications, including adjusting the content to ensure it can be delivered even if the patient is not fully aware of cancer diagnosis and delivering the first session face-to-face with flexibility for subsequent sessions.

## Background

Palliative care improves outcomes in patients with advanced cancer, especially when provided early in the disease trajectory [1–4]. Key content elements of early palliative care that have been associated with improved patient outcomes are those that focus on developing better coping skills, navigating treatment decisions, and engaging in advance care planning [5, 6]. The ENABLE (Educate, Nurture, Advise, Before Life Ends) telehealth model was developed specifically to address the need for early integration of palliative care in patients with advanced cancer and their family caregivers [7, 8]. The ENABLE model is delivered by a nurse coach who guides patients and caregivers in principles of problem-solving, coping, decision-making, advance care planning, symptom management, self-care, communication, and life outlook and review [9]. Each session starts with screening for problems using the distress thermometer; any specific problems identified could also be addressed. Table 1 shows the content outline of the ENABLE sessions. When provided to individuals with newly diagnosed advanced cancer, clinical trials of ENABLE have demonstrated better quality of life and mood, with additional benefits of earlier initiation on patient survival and family caregiver mood and burden [10, 11].

**Table 1:** Content outline of ENABLE sessions

Outline of topics in the original ENABLE model
**ENABLE sessions for patients and caregivers**
Session 1: Handling problems with a positive attitude
- COPE: A positive problem-solving attitude
- The seven steps of problem-solving
Session 2: Taking care of you
- Healthy eating, nutrition, medication and exercise
- Getting the support you need
Session 3: Taking control of your symptoms
- Common symptoms in cancer
- Spirituality
Session 4: Talking about what matters most and making choices
- Communicating with your family and healthcare providers
- Core values: what matters most
- Decision aids: making choices that are right for you
- Advance care planning
**Additional sessions for patients**
Session 5: Telling my life story
Session 6: Looking at today, looking at tomorrow

The ENABLE model was first developed in the geocultural context of the rural US states of New Hampshire and Vermont [7, 8]. It has since been adapted for African-American and Turkish context, and is currently being implemented in community oncology practices in the US [12–15]. While the overall goals of the ENABLE model to support patients and caregivers in their advanced cancer journey may be cross-culturally relevant, some of the content and how it is delivered may not be consistent with other cultural beliefs and practices. For example, the guiding principle of ENABLE is to encourage patient activation and self-management; however, this approach may have to be modified in the context of family-centred decision making and non-disclosure of diagnosis or prognosis to patients, which is prevalent in Singapore [16–18]. This would also have relevance in the other cultural settings where family plays a central role in health-related decision making [19–21]. Therefore, the main aim of this study was to evaluate the cultural relevance of the ENABLE model of early palliative care and to identify modifications for an adapted ENABLE-Singapore (SG) model to pilot test on patients with advanced cancer and their caregivers in Singapore. The cultural adaptation of ENABLE in Singapore will pave the way for future iterations of ENABLE for the family-centric cultural context to maximise its benefit [13, 22].

## Methods

This was a qualitative formative evaluation study, presented here in accordance with the consolidated criteria for reporting qualitative research (COREQ) guidelines [23, 24]. Semi-structured one-on-one interviews were conducted in English with patients with advanced cancer, their family caregivers and healthcare professionals to explore their views on (i) the main needs and challenges of individuals with advanced cancer and their family caregivers; (ii) patient involvement in healthcare decision making; (iii) content and delivery of the ENABLE model. This study was conducted in English as it is the working language in Singapore and 80% of the population is literate in English [25]. The study protocol was approved by the Singhealth Centralised Institutional Review Board (CIRB Reference Number 2018/2905).

### Participant selection and study setting

Participants were purposively sampled and recruited from the oncology and palliative care outpatient clinics of the National Cancer Centre of Singapore (NCCS) and Khoo Teck Puat Hospital (KTPH). NCCS is the largest provider of public cancer care in Singapore, with about 150,000 patient visits per year; KTPH is a 795-bed general and acute care hospital with palliative care outpatient clinics. Patient eligibility criteria included: (i) diagnosed with a stage IV solid tumour cancer; (ii) aged 21 years and over; (iii) able to converse in English. Family caregiver eligibility criteria included: (i) self-endorsing caring for a family member with stage IV solid tumour cancer; (ii) aged 21 years and over; (iii) able to converse in English. Patients and family caregivers were not dyads, caregivers who participated gave their perspectives on caring for a family member with advanced cancer. There was no limitation on the time since diagnosis. Eligibility criteria for healthcare professionals included: (i) main area of work is in medical oncology or palliative medicine; (ii) involved in patient care.

Attending oncology physicians first informed identified patients and caregivers about the study, invited them to participate and sought their permission to be referred to the study team. The study team then explained the study objectives further and what their participation would involve. After participants signed informed consent, interviews were conducted by research coordinators in NCCS (ARXK) and KTPH (CCY, LMK) who had prior experience in qualitative research and received further training in qualitative interviewing by the principal investigator (GMY). The interviewers were not healthcare professionals and had no prior relationship with the participants. Interviews were conducted in a private room or a suitable location chosen by the participant. An interview guide was developed with open-ended questions for one-on-one semi-structured interviews lasting 30 to 60 min (see Table 2 for sample interview questions). Interviews with participants were audio-recorded then later transcribed by ARXK. Field notes were also taken to document non-verbal cues and were read together with transcripts during analysis.

**Table 2:** Sample interview questions

Needs and Challenges
1. What do you think are the greatest challenges faced by patients/caregivers caring for patients with cancer?
2. What do you think are the biggest needs of cancer patients and/or their caregivers?
Healthcare Decision-Making
1. What healthcare decisions do cancer patients have to make/caregivers help patients make?
2. What things make it difficult for patients/caregivers to make healthcare decisions?
3. What information or assistance would be helpful to patients/caregivers as they make healthcare decisions?
Views on ENABLE Programme
1. In looking at each session’s topical content specifically, how appropriate do you feel each is? What topics do you think should be taken out? What topics do you think need to be added?
2. There would be six, 1-h weekly sessions. What do you think about the number and length of these sessions?

### Data analysis

Data were analysed using a thematic analysis approach [23]. Data from patients, family caregivers and healthcare professionals were analysed together to generate combined perspectives. As the interviewers would be involved in adapting the ENABLE material, coding was done by different members of the study team to include wider perspectives. The first phase of the coding process involved inductive coding of five transcripts independently by YHF, NH, AC, and GMY. YHF, NH and AC were medical students who were familiar with the clinical context, but had no preconceived notion of how the ENABLE model should be adapted for the Singapore context. Within the broad themes of i) needs and challenges of individuals with advanced cancer and their family caregivers; (ii) patient involvement in healthcare decision making; (iii) views on the ENABLE model, codes were identified from transcripts. Fieldnotes were used to check if codes accurately reflected the participants’ context. Coding categories were discussed, and we reached consensus on a common set of categories. Subsequently, all transcripts were separately coded by YHF, NH, AC. Initial analysis was performed on the transcripts for 5 patients, 5 caregivers and 5 healthcare professionals recruited from NCCS. Subsequent analysis was performed on the transcripts for 5 patients, 6 caregivers and 5 healthcare professionals recruited from KPTH. The data from KTPH added richness to the themes derived from the NCCS data, however no new themes were revealed in the KTPH data and thematic saturation was deemed to be met. Data analysis was an iterative process in which codes were discussed and the categories and sub-categories were adapted throughout the process. Regular discussions were held among the core study team members involved in the data analysis (YHF, NH, AC, GMY, IT, MAB, JND-O). Data saturation was reached when no new themes were identified.

## Results

Ten patients, 11 caregivers, and 10 healthcare professionals participated and gave their perspectives on (i) needs and challenges of advanced cancer patients and their caregivers; (ii) patient involvement in decision-making; (iii) content and delivery of the ENABLE model (Table 3). The main themes, illustrative quotes, and proposed modifications to the ENABLE model based on the themes are summarized in Table 4.

**Table 3:** Participant characteristics

**ID**	**Gender**	**Age**	**Race**	**Primary diagnosis of patient/ professional role**
**PT- Patient**				
PT01	F	80	Chinese	Colon cancer
PT02	M	41	Filipino	Ceacal cancer
PT03	F	38	Chinese	Colon cancer
PT04	M	79	Chinese	Stomach cancer
PT05	M	65	Chinese	Colon cancer
PT102	M	73	Chinese	Lung cancer
PT106	F	48	Chinese	Breast cancer
PT107	M	63	Indian	Lung cancer
PT108	M	87	Chinese	Prostate cancer
PT113	F	80	Eurasian	Colon cancer
**CG—Caregiver**				
CG01	M	68	Chinese	Rectosigmoid cancer
CG02	M	72	Chinese	Lung cancer
CG03	F	40	Chinese	Stomach cancer
CG04	M	27	Chinese	Pancreatic cancer
CG05	F	42	Malay	Stomach cancer
CG109	F	62	Chinese	Lung cancer
CG110	F	61	Chinese	Kidney cancer
CG111	M	62	Chinese	Intestinal cancer
CG112	F	Unknown	Chinese	Intestinal cancer
CG114	F	46	Malay	Colon cancer
CG115	M	30	Chinese	Lung cancer
**HW- Healthcare Professional**				
HW01	F	50	Chinese	Medical Social Worker
HW02	F	43	Chinese	Nurse Clinician (Oncology- Breast)
HW03	M	40	Indian	Senior Consultant (Oncology)
HW04	F	39	Chinese	Consultant (Palliative Medicine)
HW05	F	44	Chinese	Senior Nurse Clinician/ Advanced Practice Nurse (Palliative Medicine)
HW100	F	32	Chinese	Occupational Therapist
HW101	F	31	Chinese	Medical Social Worker
HW103	F	32	Chinese	Dietician
HW104	F	41	Chinese	Palliative Medicine Physician
HW105	F	32	Chinese	Psychologist

**Table 4:** Main themes, illustrative quotes, and intervention modifications

***Theme***	***Sample Quotes***	***Implication for ENABLE-SG model***
Needs and Challenges		
Patients experience physically challenging symptoms that also stress family	“…[patients] can't do a lot of the housework they [used] to do, or even taxi drivers they can't engage in driving…” (HW01)	• Identify cancer symptoms and side effects of treatment that are specific and relevant to the patient, and tailor advice accordingly, so that patients and caregivers know what to expect and how to manage symptoms • Advise on symptom red flags to look out for and how to seek emergency help
	“… some side effects that we (caregivers) wouldn't know how to deal with it… but… you just have to learn as you go along so it is not so easy” (CG03)	
Patients and caregivers experience psycho-emotional struggles	“…[patients] struggle with… loneliness… mental issues, dealing with the sadness, inability to function like they used to, leading to depression” (HW03)	• Provide strategies to help patients and caregiver stay positive • Provide strategies for open communication between patients and caregivers to address emotion masking • Promote self-care for caregivers • Provide list of resources and support to deal with mental health issues • Advise how to monitor distress and when to seek professional help
	“I cannot cry in front of [the patient]…” (CG05)	
	“…a lot of [caregivers’] time and effort …is all [engaged] with the patient, [caregivers] don't have much time to even care for themselves” (HW01)	
	“[caregiving information] is all quite medical but not necessary and it's all about external, there's not a lot about how do you handle the emotions when they break down what do you do, nobody give you any guidance on that…” (CG03)	
Psycho-emotional support is often offered too late	“… physical symptoms are more complicated [hence] the priority is [for] the physical symptoms to settle first… that doesn't give you much space and time to deal with the psycho-emotional parts” (HW01)	• Need to identify at-risk population and offer support earlier in disease trajectory
Patient involvement in healthcare decision making		
Preference for decision making (patient-led vs doctor-led vs shared) was varied among participants	“… the family were all called to listen to [the treatment options] and then come to a conclusion…” (CG111 and CG112)	• Identify preferred decision-making model and family dynamics so that advice to patients and caregivers can be tailored accordingly
	“…patients who are very independent… want things… to be done [a] certain way… they do not want caregivers to be involved at all” (HW02)	
	“…[the decision was] still very much geared up to what the doctor thinks is the best thing to do [is]” (CG03)	
	“… [the doctor] knows best what my condition is… so I just do [the chemotherapy]” (PT04)	
Diagnosis disclosure not culturally appropriate	“[I] rather [not] tell [the patient] the ‘big C word’” because that would be “[taking] away that hope… [of] the chance to cure” (CG109)	• Assess patient and caregiver preferences for disclosure of diagnosis and prognosis • Check if family are willing for participation in ENABLE • Focus on content that does not require awareness of advanced cancer stage and/or prognosis
Content of ENABLE		
Advance care planning (ACP) discussions require good rapport	“…[advance care planning] will allow patients to be able to share about their personal values, their beliefs, preferences…” (HW02)	• Bring up ACP later on in the programme, when more rapport has been established
	“If you have not build rapport with the patient yet and they are just coming to terms with their diagnosis I think advanced care planning may be just a bit too much to handle cause they don't know where they are headed now… later on maybe after third or fifth follow-up…then it's fine to bring it up…” (HW03)	
Financial matters are a concern for patients and families	“…[patients] are also afraid of… what kind of burden [the illness] has upon the family members, and especially if they are… the sole breadwinner of the family, then who is going to take over the role of getting money for the family…” (HW01)	• Provide information on financial matters and financial aid early on
Body image and sexuality is an issue for some patients	“I experience this part (changes in sexuality) as well because there was a severe change… for women it can be demoralising… especially those patients that are undergoing let's say breast cancer…” (CG03)	• Provide opportunity to discuss changes in body image, sexuality and fertility
	“I feel like this topic … would be a difficult topic for them to talk about…whether will they even be open to discussing that kind of topic with healthcare providers about their relationship with their spouse and how they can you know, kind of regain back their intimacy.” (HW02)	
Delivery of ENABLE		
Avenue for asking questions in between sessions	“Maybe there should be a hotline for the patients to call you so when they have any issues, they can actually call the hotline to make some enquiries.” (PT03)	• Give patients and caregivers a point of contact to reach the ENABLE nurse coach
	“an email kind of thing where they can, feedback with some questions, if after their consult, their talks, their face-to-face and over the phone. Maybe some, other things might come out for them, so is there another room for them to ask in between those days.” (HW01)	
Frequency and duration of sessions to be kept flexible	“it also depends on the patient himself or herself in terms of the independence mobility … if they … need someone to bring them and whether the caregiver can afford the time as well… for the duration patients might need to be given a break they are very lethargic it might be good to keep it a bit shorter versus patients who can do a bit more.” (HW105)	• Allow flexibility in scheduling of ENABLE sessions
	“we have to really do consider just working around with patient's schedule.” (HW02)	
Face-to-face sessions may be more effective	“you can get instant feedback and then you know whether this person is receptive to what you suggest or whether this person has any other questions, that they probably may not say over the phone.” (HW100)	• Conduct the first ENABLE session face-to-face, and allow flexibility for subsequent sessions to be conducted over the phone or face-to-face

### Needs and challenges

Physical symptoms from the disease and the treatment side effects were cited as the main challenges faced by advanced cancer patients. Physical problems such as pain, nausea and fatigue affected their ability to work and function in their usual activities of daily living. *“The side effects have been very severe and I would say the side effects give me a lot of problems like giddiness, fingers are numb and the skin is either very dry or can be easily hurt if I knock against the wall.” (PT04)* Caregivers expressed a sense of helplessness at not knowing how to manage these physical symptoms and felt that more information on what to expect and how to manage them would be helpful *“to understand, how do we manage her and all that.” (CG111)*

Patients and caregivers also experienced psycho-emotional challenges. Initially, patients felt shock and fear experiencing cancer *“like a death sentence” (HW01, CG01)*. *“When you discover you have cancer, everything falls down, your mind becomes blank.” (PT102)* During the course of treatment, some patients felt anxious about treatment while others felt *“demoralised” (PT03)* by how their daily life was affected by the cancer and the treatment side effects.

Caregivers felt *“stressed from caring for another person” (HW04)*, and felt *“burnout”* related to the *“multiple roles… caring for their own family, caring for their own children at home, and then still having to take care of the patient undergoing treatment” (HW02)*. However, caregivers felt the need to mask their emotions as they felt that the patient’s needs had to take precedence. *“A lot of attention has to be given to [the patient] … my routine work, meeting with friends for exercise – if he has an appointment, I have to cancel all these.” (CG01)*

Participants reported that in comparison to receipt of support for physical needs, *“psychosocial and emotional support is offered too late in the trajectory, when social workers don’t have enough time to build rapport with the patient.” (HW01)* Also, there might be *“stigma” (HW02)* associated with seeing a social worker or psychologist, and patients may feel more comfortable seeking psycho-emotional support from nurses instead.

### Patient involvement in healthcare decision making

Participants reported a variety of decision-making styles. For example, regarding family involvement, one patient shared “*I told my children: I make my own decisions. You all don’t make decisions for me.” (PT113)*; on the other hand, another patient shared *“I always talk about it with my wife. And then basically we’re the ones who make the decisions, there’s no one else.” (PT02)*. Yet others expressed a medically-guided decision making approach: *“It is not easy just to make your own decision about medical treatment because you are not in it, so you don’t know anything about cancer, you may not know what to do” (CG111)* As the doctor is the medical expert, *“whatever the doctor says, we must follow the doctor’s decision.” (CG01)* Regardless of decision-making preference, it is important for the doctor to share information with patients and their families so that *“they can understand what is going on.” (HW01)*

There were also several instances when family are concerned about disclosing the cancer diagnosis and/or prognosis to the patient, particularly with older adults, due to concerns that *“they can’t take it” (HW01)*. Sometimes, patients themselves ceded decision making to their family, for example one patient shared *“I have given the go for my brother and daughter to decide for me” (PT102)*.

### Content of ENABLE model

The content of ENABLE was mostly felt to be *“relevant”, “comprehensive” (HW03)*, and *“practical” (HW04)* in the Singapore cultural context. Advance care planning was identified as an important topic, but the session has to be *“tailor-made” (CG03)* for the individual patient’s circumstances and the conversation needs to be navigated sensitively: *“So I think this is a very important topic, but how to bring it up would very much depend on how this nurse could actually bring it up” (HW02)*. Additional topics suggested included how to evaluate complementary therapy options such as acupuncture and traditional Chinese medicine, dietary advice, and information on sources of practical caregiver support.

Financial and sexuality concerns were two additional topics suggested by participants and explored further in the study interviews. *“Patients will often ask for financial information but it might often stop at how much a treatment costs at that point in time, not what would happen or what you might need or if you cannot work what is the impact…” (HW04)* Part of the worry is the uncertainty regarding *“how long is this period going to last and how much we are going to spend. We don’t know that.” (CG100)* Loss of income, cost of treatment and impact on family expenses were examples of financial worries cited, but participants did not suggest additional content beyond provision of information on sources of financial assistance. While sexuality was raised as a salient issue for younger patients, some felt that it was taboo to address it routinely in the context of the ENABLE model. *“Sexuality is more sensitive. Not every patient or caregiver might bring it up so we might have to ask sensitively probably for certain cancers which affect body image specifically, and touch on that earlier rather than later in the trajectory.” (HW04)*

### Delivery of ENABLE model

Participants had varied opinions on the format and delivery of ENABLE. There were suggestions for having a means of contact with the ENABLE nurse coach in between sessions and allowing for flexibility in the frequency and duration of sessions. There was a preference for sessions to be conducted face-to-face to allow nurse to better assess the patient’s or caregiver’s reaction, because *“quite personal topics” (HW105)* will be discussed. *“For phone, the coach would not be able to observe the facial expressions that the patient or caregiver has so it is very hard for us to check in if they really fully understand. And very hard for us to know whether they are comfortable to receive this knowledge or information at this moment.” (HW101)*. Face-to-face sessions were also perceived to reduce distractions: *“Every time I call patient, it seems like when they are outside or just like other things going on or maybe the background noise is very noisy. It is very hard to do the session.” (HW103)* However, participants also acknowledged there may be logistic challenges in terms of timing and travel: while it may be *“great if the session can be on the same day”* to reduce the need for additional travel, it can also be quite *“taxing”* for the patient and caregiver to spend a long time at the healthcare facility. *(HW103)*

## Discussion

The main aim of this formative evaluation trial was to assess the perspectives of patients, caregivers, and clinicians in Singapore on the needs and challenges in the setting of a new diagnosis of advanced cancer, and cultural relevance of the ENABLE model. Overall, the ENABLE model was perceived to be relevant and useful in our cultural context. Nonetheless, our study findings point to three main areas of modification to create an adapted ENABLE-SG for pilot testing.

First, the results identified thematic areas that are salient to advanced cancer patients and their family caregivers. Besides physical problems arising from the disease as well as cancer treatment, a particular area of unmet need that emerged was psychosocial and emotional support for patients and caregivers [26–28]. Aspects of psychosocial support such as problem-solving, self-care, communication strategies and navigating treatment decisions are already in the original ENABLE model (Table 1) – these will be retained in the adapted ENABLE-SG. Advance care planning was specifically raised as a topic that should be addressed in later sessions when more rapport has been established. In the original ENABLE model, this is introduced in session 4 “Talking about what matters most and making choices” and will be kept here as the nurse coach should have built sufficient rapport by this time. However, there will also be flexibility to defer advance care planning to a later session if the nurse coach judges that the patient is not ready.

Additional areas of concern include financial needs, evaluation of complementary therapy, dietary advice, body image and sexuality changes. These will be added as optional topics for discussion in session 2 “Taking care of you”. For financial needs, the distress thermometer used to screen for problems at the start of each session will contain an item for financial concerns, triggering a referral to existing avenues of financial assistance if required. In the original ENABLE model, the nurse coach would screen for these topics and discuss them in greater detail if these were areas of concern to patients or caregivers. However, participants in this study seemed to desire additional written information on these topics, which could also be included in other iterations of ENABLE worldwide.

Second, in a family-centric cultural context, patients may not be fully aware of their cancer diagnosis and prognosis. This family-centred decision-making approach is prevalent in many cultures worldwide [29, 30]. Families may feel it is their responsibility to receive bad news and decide how much should be disclosed to the patient, so as to protect the sick patient from unnecessary worry and to preserve hope; there is also a cultural obligation for the family member to bear the burden of making decisions on behalf of the patient [30]. In the context of ENABLE which seeks to help patients and family caregivers cope with problems arising from advanced cancer and/or cancer treatment, participants should at least be aware of their cancer diagnosis. However, in order to ensure that healthcare interventions are aligned with family-centric cultural values, the content of ENABLE-SG should be appropriate for patients who do not know the full extent of their cancer and where decisions are delegated to a family member. Sessions 1 to 4 of the original ENABLE model focus on coping skills that are applicable at all stages of cancer, and will only require minor modifications so that they can be conducted for patients who are aware of their cancer diagnosis but not their prognosis. For example, in the section on healthy eating and nutrition, content on how diminished appetite can be “due to advancing illness or inability to control the cancer” could be removed. Sessions 5 and 6 on life review and creating a legacy may be challenging to address if the patient is not aware of their advanced stage of cancer. However, the nurse coach would have developed sufficient rapport with the patient by that time to judge whether it would be appropriate to proceed with these sessions sensitively, or defer them to a subsequent monthly follow-up call when the opportunity arises.

Notwithstanding the central role of the family in medical decision making, various decision-making styles were represented in our sample, including patient-directed and shared decision-making approaches [31]. While some patients would defer treatment discussions to their family, others in the same geographical context may advocate for an informed and activated patient who has productive interactions with the healthcare team, and adopt a shared decision-making approach [32]. Therefore, healthcare decision making approaches need to be tailored to individual family dynamics in the ENABLE-SG model. The content of session 4 “Talking about what matters most and making choices” will be modified to include an additional section on talking about decision-making preferences. The nurse coach will also be trained to continually assess preferred decision-making preferences and deliver the ENABLE-SG sessions accordingly.

Third, there was a preference for face-to-face sessions to allow for the use of non-verbal communication to strengthen the effectiveness of the model. This is particularly salient given the need to assess patient and caregiver preferences for information disclosure and decision-making approaches. Therefore, the adapted ENABLE-SG model will be delivered face-to-face at least for the first session to facilitate rapport building, with flexibility in subsequent sessions being conducted face-to-face or over the phone. Flexibility in mode of delivery of ENABLE-SG will be helpful to comply with physical distancing measures in the context of the ongoing Covid-19 pandemic.

A limitation is the study sample may not be entirely representative of the wider population of oncology patients, caregivers and healthcare professionals in Singapore. Specifically, patients who are not fully aware of their diagnosis may be unrepresented, although we sought to elicit the perspectives of these patients through interviews with healthcare professionals. However, we used purposive sampling to ensure that participants represented the range of demographic characteristics, primary cancer type and professional backgrounds, while focusing on stakeholders for our target population of advanced cancer patients and caregivers. Therefore, we elicited a broad spectrum of perspectives to inform the cultural adaptation of ENABLE-SG for further testing.

## Conclusions

To our knowledge, this is the first study to evaluate the relevance and suitability of ENABLE in the Southeast Asian geographical context. This could be a model for other countries in the region to implement their own versions of the ENABLE model. Our findings show that ENABLE is broadly relevant to the Singapore context, however it requires some cultural adaptations to optimise its effectiveness in the local population. Three main modifications are suggested to the content and delivery of ENABLE – (i) include additional topics for discussion if relevant to the patient or family; (ii) assess preferences for decision making and tailor content to cater for individual family dynamics; (iii) deliver the first session face-to-face if possible and allow flexibility in mode of delivery for the rest of the sessions. Our findings will be used to guide the future pilot testing of the ENABLE-SG model in order to further evaluate the feasibility and acceptability using the input of our stakeholders and continue to iterate a culturally sensitive and effective model of early palliative care for patients with advanced cancer and their family caregivers.

## Data Availability

The data of this study are stored at the National Cancer Centre of Singapore. Data are available upon reasonable request to the corresponding author.

## Abbreviations

ENABLE: Educate, Nurture, Advise, Before Life Ends
NCCS: National Cancer Centre of Singapore
KTPH: Khoo Teck Puat Hospital

## References

[1] Gaertner J, Siemens W, Meerpohl JJ, Antes G, Meffert C, Xander C, Stock S, Mueller D, Schwarzer G, Becker G (2017). Effect of specialist palliative care services on quality of life in adults with advanced incurable illness in hospital, hospice, or community settings: systematic review and meta-analysis. BMJ.

[2] Haun MW, Estel S, Rucker G, Friederich HC, Villalobos M, Thomas M, Hartmann M (2017). Early palliative care for adults with advanced cancer. Cochrane Database Syst Rev.

[3] Hui D, Bruera E (2020). Models of palliative care delivery for patients with cancer. J Clin Oncol.

[4] Nickolich MS, El-Jawahri A, Temel JS, LeBlanc TW (2016). Discussing the evidence for upstream palliative care in improving outcomes in advanced cancer. Am Soc Clin Oncol Educ Book.

[5] Hoerger M, Greer JA, Jackson VA, Park ER, Pirl WF, El-Jawahri A, Gallagher ER, Hagan T, Jacobsen J, Perry LM (2018). Defining the elements of early palliative care that are associated with patient-reported outcomes and the delivery of end-of-life care. J Clin Oncol.

[6] Maloney C, Lyons KD, Li Z, Hegel M, Ahles TA, Bakitas M (2013). Patient perspectives on participation in the ENABLE II randomized controlled trial of a concurrent oncology palliative care intervention: benefits and burdens. Palliat Med.

[7] Bakitas M, Lyons K, Hegel M, Balan S, Brokaw F, Seville J, Hull J, Li Z, Tosteson T, Byock I (2009). Effects of a palliative care intervention on clinical outcomes in patients with advanced cancer: the Project ENABLE II randomized controlled trial. JAMA.

[8] Bakitas M, Stevens M, Ahles T, Kirn M, Skalla K, Kane N, Greenberg ER (2004). Project ENABLE: a palliative care demonstration project for advanced cancer patients in three settings. J Palliat Med.

[9] Bakitas M, Lyons KD, Hegel MT, Balan S, Barnett KN, Brokaw FC, Byock IR, Hull JG, Li Z, McKinstry E (2009). The project ENABLE II randomized controlled trial to improve palliative care for rural patients with advanced cancer: baseline findings, methodological challenges, and solutions. Palliat Support Care.

[10] Bakitas MA, Tosteson TD, Li Z, Lyons KD, Hull JG, Li Z, Dionne-Odom JN, Frost J, Dragnev KH, Hegel MT (2015). Early versus delayed initiation of concurrent palliative oncology care: patient outcomes in the ENABLE III randomized controlled trial. J Clin Oncol.

[11] Dionne-Odom JN, Azuero A, Lyons KD, Hull JG, Tosteson T, Li Z, Li Z, Frost J, Dragnev KH, Akyar I (2015). Benefits of early versus delayed palliative care to informal family caregivers of patients with advanced cancer: outcomes from the ENABLE III randomized controlled trial. J Clin Oncol.

[12] Akyar I, Dionne-Odom JN, Ozcan M, Bakitas MA (2019). Needs assessment for Turkish family caregivers of older persons with cancer: first-phase results of adapting an early palliative care model. J Palliat Med.

[13] Akyar I, Dionne-Odom JN, Yang GM, Bakitas MA (2018). Translating a US early palliative care model for Turkey and Singapore. Asia Pac J Oncol Nurs.

[14] Hendricks BA, Lofton C, Azuero A, Kenny M, Taylor RA, Huang CS, Rocque G, Williams GR, Dosse C, Louis K (2019). The project ENABLE cornerstone randomized pilot trial: protocol for lay navigator-led early palliative care for African-American and rural advanced cancer family caregivers. Contemp Clin Trials Commun.

[15] Zubkoff L, Lyons KD, Dionne-Odom JN, Hagley G, Pisu M, Azuero A, Flannery M, Taylor R, Carpenter-Song E, Mohile S (2021). A cluster randomized controlled trial comparing Virtual Learning Collaborative and Technical Assistance strategies to implement an early palliative care program for patients with advanced cancer and their caregivers: a study protocol. Implement Sci.

[16] Back MF, Huak CY (2005). Family centred decision making and non-disclosure of diagnosis in a South East Asian oncology practice. Psychooncology.

[17] Lee GL, Teo I, Kanesvaran R (2018). The complexities of doctor–patient–family communication in an Asian oncology setting: concordance and discordance among patient preferences, family preferences, and perceived and actual communication. Health Commun.

[18] Tay K, Yu Lee RJ, Sim SW, Menon S, Kanesvaran R, Radha Krishna LK (2017). Cultural influences upon advance care planning in a family-centric society. Palliat Support Care.

[19] Blackhall LJ (1995). Ethnicity and attitudes toward patient autonomy. JAMA.

[20] Hobbs GS, Landrum MB, Arora NK, Ganz PA, Van Ryn M, Weeks JC, Mack JW, Keating NL (2015). The role of families in decisions regarding cancer treatments. Cancer.

[21] Lee SK, Knobf MT (2016). Family involvement for breast cancer decision making among Chinese-American women. Psychooncology.

[22] Dionne-Odom JN, Taylor R, Rocque G, Chambless C, Ramsey T, Azuero A, Ivankova N, Martin MY, Bakitas MA (2018). Adapting an early palliative care intervention to family caregivers of persons with advanced cancer in the rural deep south: a qualitative formative evaluation. J Pain Symptom Manage.

[23] Patton M (2015). Qualitative research and evaluation methods.

[24] Tong A, Sainsbury P, Craig J (2007). Consolidated criteria for reporting qualitative research (COREQ): a 32-item checklist for interviews and focus groups. Int J Qual Health Care.

[25] Department of Statistics, Ministry of Trade & Industry, Republic of Singapore. Census of population 2010 statistical release 1. 2010.

[26] Mitchell AJ, Chan M, Bhatti H, Halton M, Grassi L, Johansen C, Meader N (2011). Prevalence of depression, anxiety, and adjustment disorder in oncological, haematological, and palliative-care settings: a meta-analysis of 94 interview-based studies. Lancet Oncol.

[27] Wang T, Molassiotis A, Chung BPM, Tan JY (2018). Unmet care needs of advanced cancer patients and their informal caregivers: a systematic review. BMC Palliat Care.

[28] Wang T, Molassiotis A, Tan JY, Chung BPM, Huang HQ. Prevalence and correlates of unmet palliative care needs in dyads of Chinese patients with advanced cancer and their informal caregivers: a cross-sectional survey. Support Care Cancer. 2020. 10.1007/s00520-020-05657-w 32776164

[29] Chong JA, Quah YL, Yang GM, Menon S, Radha Krishna LK (2015). Patient and family involvement in decision making for management of cancer patients at a centre in Singapore. BMJ Support Palliat Care.

[30] Krishna LK, Alsuwaigh R, Miti PT, Wei SS, Ling KH, Manoharan D (2014). The influence of the family in conceptions of personhood in the palliative care setting in Singapore and its influence upon decision making. Am J Hosp Palliat Care.

[31] Khosla N, Washington KT, Mukherjea A, Aslakson R (2019). Health-care providers’ perspectives on decision-making among seriously ill patients of south Asian origin in the United States. J Palliat Care.

[32] Malhotra C, Kanesvaran R, Barr Kumarakulasinghe N, Tan SH, Xiang L, Tulsky JA, Pollak KI (2020). Oncologist-patient-caregiver decision-making discussions in the context of advanced cancer in an Asian setting. Health Expect.

